# An Introduction to Pathology and Morbid Anatomy

**Published:** 1872-05

**Authors:** 


*An Introduction to Pathology and Morbid Anatomy.* By T. Henry Green, M. D., Lond. Illustrated by numerous engravings on wood. Philadelphia: Henry C. Lea, 1871.

In his Preface the author modestly disclaims any other title for his work that of an Elementary or Introductory work in morbid Anatomy; yet a cursory examination would seem to indicate that, although it does not reach the high standard of the work of Rindfleisch, still it possesses to considerable extent the requisites of a Text Book of Pathology.

The Book is devided into thirty-nine chapters and treats each subject in the order introduced clearly and to as full an extent as could be expected of a work of this character. The increasing importance which is being attached to the study of pathology calls for a work of this kind to prepare the student for the works of the masters. The illustrations, many of them are familiar being taken from Rindfleisch's work; yet not a few of them are original being taken from specimens in the author's possession. We know of no work of its character which we would sooner place in the hand of the student commencing the study of Morbid Anatomy.

